# Interface Effects of Strain-Energy Potentials on Phase Transition Characteristics of VO_2_ Thin-Films

**DOI:** 10.1021/acsomega.3c01966

**Published:** 2023-05-30

**Authors:** Jyrki Lappalainen, Matti Kangaspuoskari

**Affiliations:** Civil Engineering Research Unit, Faculty of Technology, University of Oulu, P.O. Box 8000, FIN-90014 Oulu, Finland

## Abstract

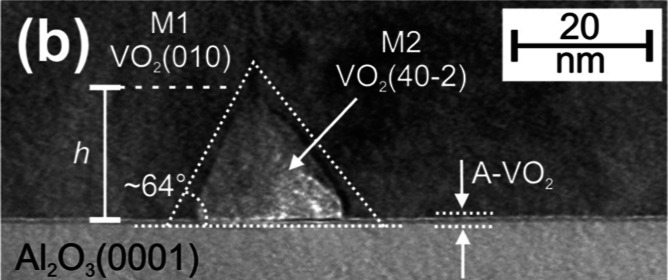

Metal–insulator-transition (MIT) of VO_2_ has attracted strong attention as a potential phenomenon to be utilized in nanostructured devices. Dynamics of MIT phase transition determines the feasibility of VO_2_ material properties in various applications, for example, photonic components, sensors, MEMS actuators, and neuromorphic computing. However, conventional interface strain model predicts the MIT effect accurately for bulk, but fairly for the thin films, and thus, a new model is needed. It was found that the VO_2_ thin film–substrate interface plays a crucial role in determining transition dynamics properties. In VO_2_ thin films on different substrates, coexistence of insulator-state polymorph phases, dislocations, and a few unit cell reconstruction layer form an interface structure minimizing strain energy by the increase of structural complexity. As a consequence, MIT temperature and hysteresis of structure increased as the transition enthalpy of the interface increased. Thus, the process does not obey the conventional Clausius–Clapeyron law anymore. A new model is proposed for residual strain energy potentials by implementing a modified Cauchy strain. Experimental results confirm that the MIT effect in constrained VO_2_ thin films is induced through the Peierls mechanism. The developed model provides tools for strain engineering in the atomic scale for crystal potential distortion effects in nanotechnology, such as topological quantum devices.

## 1 Introduction

Development of conventional microelectronics is about to reach its physical integration limits. Future technologies require a totally different perspective in manufacturing technologies, such as lithography, and materials in order to increase information processing capacity and device performance as it would be estimated by Moore’s law. Vanadium dioxide VO_2_ is a good example of so-called functional electroceramic materials, which possess particular native properties, and functions of its own to be exploited in breaking the famous red brick wall on the way of More-than-Moore concept devices. Metal–insulator-transition (MIT) effect of VO_2_ vanadium oxide has attracted strong attention as one candidate of such new materials for nanostructured devices required for integration beyond present CMOS technology, such as logic gates and memory cells.^1,2^ Also, the functional properties of vanadium oxide has been found very promising in various other applications, such as electro-optic devices, thermo-electric sensors, light modulators and electro-chromic electrodes and windows, MEMS actuators, neural and neuromorphic networks and circuits, phononics, etc., including advanced future technologies.^3−6^

All the materials processed in the form of thin-films and nanostructures on surfaces of the substrates are strongly influenced by the substrate and quantum confinement effects. The characteristic properties of the material are strongly dependent on the nature of the exact structure of the material–substrate interface, and they might divert from the bulk crystal properties in a significant way. For example, microstructure, including the type and degree of crystallinity, e.g., amorphous, polycrystalline, columnar, epitaxial, single crystal, or crystal symmetry and orientation, grain and domain size, and chemical stoichiometry are some of the material parameters that might depend strongly on the nature of interface, and consequently, have direct effects on the functionality of the thin-film material in terms of electrical conductivity, electric polarization, magnetization, optical properties, etc. Thus, from the conventional engineering point of view, the substrate and quantum confinement effects are often seen as impairment and obstacles in microelectronics integration process, where materials single-crystal bulk properties are desired to be exploited in the nanostructured thin-film devices. For most of the applications, a heteroepitaxial thin-film structure with lattice coherence across the film–substrate interface is required, and even then, inconsistencies lead to distortions, for example, due to lattice misfit strain ε, which in turn, can lead to point defects and dislocation, impairing the thin-film properties.^7,8^ This is especially pronounced in numerous semiconductor components, including lasers, diodes, advanced solar cells, etc. Thus, for various applications one can determine the highest allowed threading dislocation density (TDD) limit, like TDD < 10^9^ cm^–2^ for deep-UV optoelectronics in order to achieve the desired device functionality and performance.^9^ On the other hand, as the knowledge and fabrication methods of heteroepitaxial structures have developed during the decades, the possibility of precise control of the interface structure offers a method called strain engineering, in which the anomalous conditions in the film–substrate interface are utilized in the fabrication of totally new types of material. One good example of such a metamaterial generated through controlled strain engineering is ferroelectric SrTiO_3_ at room temperature. Thin films of SrTiO_3_, with a thickness of few tenths of nanometers, were deposited epitaxially on single-crystal DyScO_3_ substrates, where the misfit strain distorts the otherwise paraelectric phase of SrTiO_3_ in to a ferroelectric symmetry.^10^ Strain engineering can also be exploited to manipulate the otherwise nonexistent electro-optic properties of silicon by breaking the inversion symmetry in strained silicon layers in SOI structures with Si_3_N_4_ thin films.^11^ Obviously, various effects and anomalies in materials behavior and properties, even including the totally new discoveries of previously known functionalities, can actually be related to the most important and significant defect of the material, namely, the surface. When the surfaces of the two materials are brought to the vicinity of each other in atomic “galvanic” level forming electronic bonds, the discontinuity of the materials properties generate interesting new features to the interface system to be revealed. When such an interface is fabricated with a method capable to control the structure at an atomic level, it is possible to use electronic orbital occupation differences, defect chemistry and stoichiometry, and crystal potential, as well as dimensional distortions generating strain-energy potentials, across the interface to introduce two-dimensional topological insulators and quantum phenomena, such as electron gas (2DEG) and skyrmions, for example. Several new future technologies can be envisaged based on these phenomena.^12−14^ In the case of vanadium oxide VO_2_, the dependence of the MIT effect on the strain state of the crystal is typically related to the strain along the *a*-axis of insulating M1 monoclinic phase, i.e., ε_*a*M1_, which is considered as the thermodynamic order parameter for first order metal–insulator phase-transition to tetragonal metal phase. The underlying mechanism of this transition lays in the lattice distortions induced by some external force, such as thermal expansion, mechanical force, or electrostrictive effect, leading to new electronic occupation densities and energy band structure through V 3d orbital splitting. On the other hand, the strain is very well known to induce polymorph phases, e.g., M1, M2, and A, in to insulating the VO_2_ crystal before the transition to tetragonal metal phase occurs. These polymorph phases were also found to form ferroelastic twin domain structures in order to adopt the VO_2_ crystal structure to the elastic strain. Thus, the theory of MIT effect is still under a constant debate, and includes both pure electron-correlation contribution in the form of Mott–Hubbard model and also structural phase transition (SPT) contribution according the Peierls theory. Experiments have also shown that several routes and scenarios are possible for MIT process, through various phases, depending on the parameters, such as specimen structural details, temperature, strain, etc.^15−17^

Heteroepitaxial material interface is the most important part of almost every semiconductor device, and thus, the present state of theory and engineering of the structure at atomic level are already very advanced. Nevertheless, the elastic misfit strain in the congruent epitaxial interface still remains often as the limiting device performance factor, as well. Fundamental descriptions of relationship between mechanical misfit strain and defect formation, such as dislocations, in the film–substrate interface have been completed with modern methods, for example, applying phase-field crystal models for dynamic thin-film growth process simulations, etc. Surprisingly enough, it is still found that the experimentally determined dislocation densities are typically clearly lower than predicted by the models.^18−20^ In this paper, we present a modified dislocation density model for a static two-dimensional VO_2_ thin film–substrate interface structure according to the various experimental properties determined for insulator state VO_2_ at the room temperature. Total residual elastic energy *E*_ε_ is calculated, and the effects of dislocations and polymorph phases found in the VO_2_ thin film–substrate interface are included in the model. The capped layer approximation was used for modeling the TD created in the VO_2_ thin film–substrate interface for two reasons. The initial strain values were found very high, |ε_0_| > 2.5%, which typically infers to critical thickness values comparable to length of Burger vector, i.e., unit cell length in this case, capable of generating dislocations even in to extremely thin films. On the other hand, polymorphic phase transitions could be implemented directly in to the model simply by matrix presentations. Presented new model was found to explain explicitly the dependence of *T*_MIT_ and hysteresis behavior due to complex interface structure.^21^

## 2 Experimental Section

In contrary to many other research reports, in this paper, we select the perspective which pronounces the effects of different types of the VO_2_ thin film–substrate interfaces through the strain and microstructure effects on MIT properties, rather than considering the effects of the thickness-dependent strain state on the thin-film characteristics.^16^ Definitely, we also considered the effect of thickness on MIT behavior among the samples studied here, but as described later in detail, the film thickness was not really the dominating factor. All of the samples were grown using the in situ pulsed laser deposition (PLD) method in similar conditions and parameters in order to obtain stoichiometric chemical composition of highly oriented epitaxial VO_2_ thin films on all of the substrates studied.

For the depositions, a Lambda-Physic COMPex 201 XeCl excimer laser with wavelength of 308 nm, pulse duration of 25 ns, and pulse repetition rate of 5 Hz, was used in the experiments. Laser pulse energy density of 3 J/cm^2^ was carefully adjusted on the target surface. A commercial (SCI Instruments) high-purity sintered ceramic V_2_O_5_ pellet was used as the target. The substrate temperature was kept constant at 400 °C during the deposition, and afterward cooled down at the rate of 3.3 °C/min. For in situ growth, the atmosphere of oxygen up to 1.0–1.3 × 10^–2^ mbar was added in to the vacuum chamber, initially pumped down to base pressure of 2.5 × 10^–5^ mbar. Single-crystalline substrates with four different atomic surface structures including *a*-cut (1120), *r*-cut (1102), *c*-cut (0001) Al_2_O_3_, and MgO(100) were used in the experiments. MgO(100) was selected mainly as for the reference substrate due to its different thermal expansion coefficient α_th_ and other properties. Special attention was paid for the cleaning of the substrate surfaces with alcohol ultrasound bath, rinsing, and high nitrogen (N_2_) gas-flow drying processes before the deposition.

After the deposition, the VO_2_ thin-film samples were subjected to various characterization experiments in order to confirm their electrical and MIT properties, microstructural characteristics, such as preferred orientation, phase structure and symmetries, chemical purity and stoichiometry, and strain state.

For the electrical and MIT properties’ characterization, i.e., resistivity as a function of temperature ρ(*T*), phase transition temperature *T*_MIT_, etc., the aluminum (Al) electrodes with the thickness of 200 nm were fabricated on the top of VO_2_ thin-films using a standard photolithography process and e-beam evaporation to form the lateral device architectures. Computer-controlled Memmert UFP400 low-temperature furnace, LabView controlled HP3458A multimeter, and Keithley 2612 source meter were used for the experimental setup.

Microstructure characterization was always started with X-ray diffraction (XRD) measurements, exploiting both θ–2θ and grazing incidence diffraction methods using Philips PW1380 and Bruker D8 Discover facilities. Phase identification was continued by HOBIRA Jobin Yvon LabRAM HR800 Raman spectroscopy facility operating at laser-beam wavelength of 488 nm. Detailed microstructure studies of morphologies of both sample surface and cross-section were carried out using a Zeiss Sigma scanning electron microscope and a Tecnai Spirit G2 transmission electron microscope operated at 120 kV, respectively. For the preparation of cross-sectional samples used in scanning electron microscopy (SEM) and transmission electron microscopy (TEM) experiments, the focused ion beam facility FEI Helios Nova 600 NanoLab was utilized in the processing. Chemical composition and stoichiometric analysis of the VO_2_ thin-film structures were performed using Thermo Fisher Scientific ESCALAB 250Xi X-ray photo-electron spectroscopy (XPS) facility. A special routine was implemented to characterize the chemical composition uniformity in the direction of film thickness *t*_f_. First, the initial surface of the VO_2_ thin film was cleaned by low-energy Ar^+^-ion beam, and the first XPS measurement was made. Then, high-energy Ar^+^-ion beam was used for etching the film surface by ∼2–5 nm, followed by a low-energy Ar^+^-ion beam cleaning process. After this cycle, new XPS data were collected, and then, the whole cycle was repeated. The purpose of the low-energy Ar^+^-ion beam cleaning was to remove any amorphous VO_2_ layer from the measurement point after the high-energy beam etching. XPS spectra were recorded in the range of 500–550 eV, probing especially the vanadium V 2p_3/2_, V 2p_1/2_, and oxygen O 1s energy levels. All of the collected data sets were analyzed, and the corresponding mathematical fitting and modeling procedures were carried out mostly using OriginLab Pro 2016 software package.

## 3 Experimental Results

**Figure 1:**
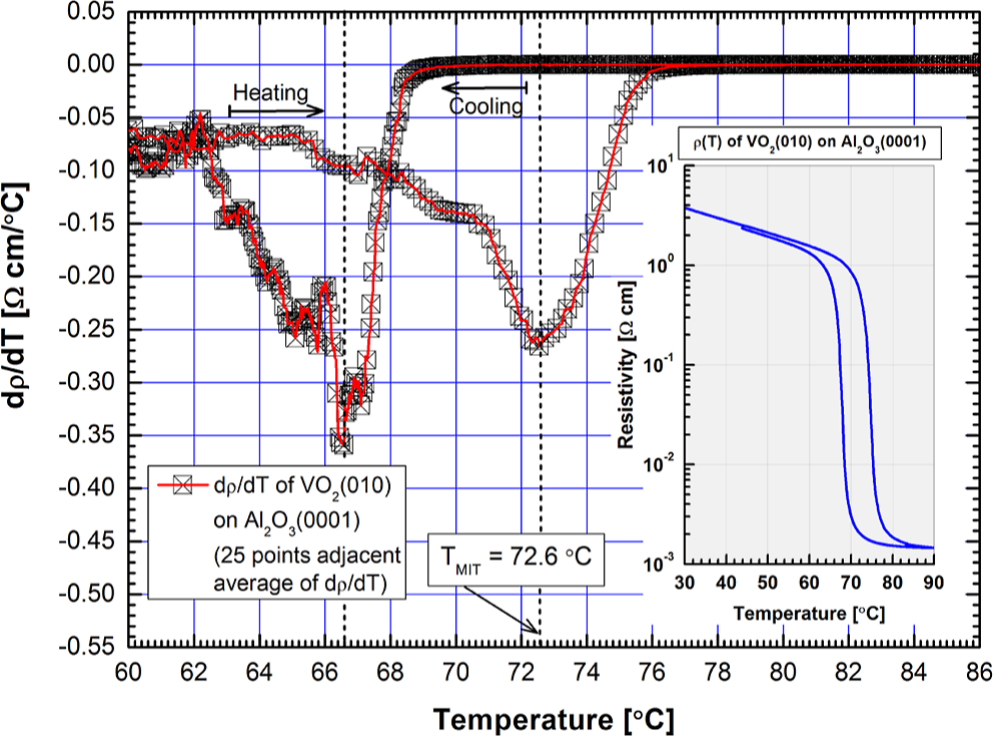
Temperature differential of electrical resistivity dρ(*T*)/d*T*, as well as a typical resistivity as a function of temperature in the inset, of a VO_2_ thin-film resistor device on *c*-cut (0001) Al_2_O_3_ substrate. MIT temperatures *T*_MIT_ for both heating and cooling cycles are pointed out by dashed vertical lines.

Properties of the MIT of VO_2_ thin films on various substrates were characterized by measuring the conventional temperature dependence of resistivity ρ(*T*). Temperature differential of electrical resistivity dρ(*T*)/d*T*, as well as a typical resistivity as a function of temperature of a VO_2_ thin-film resistor device are shown in Figure 1, and in the inset, respectively.

**Figure 2:**
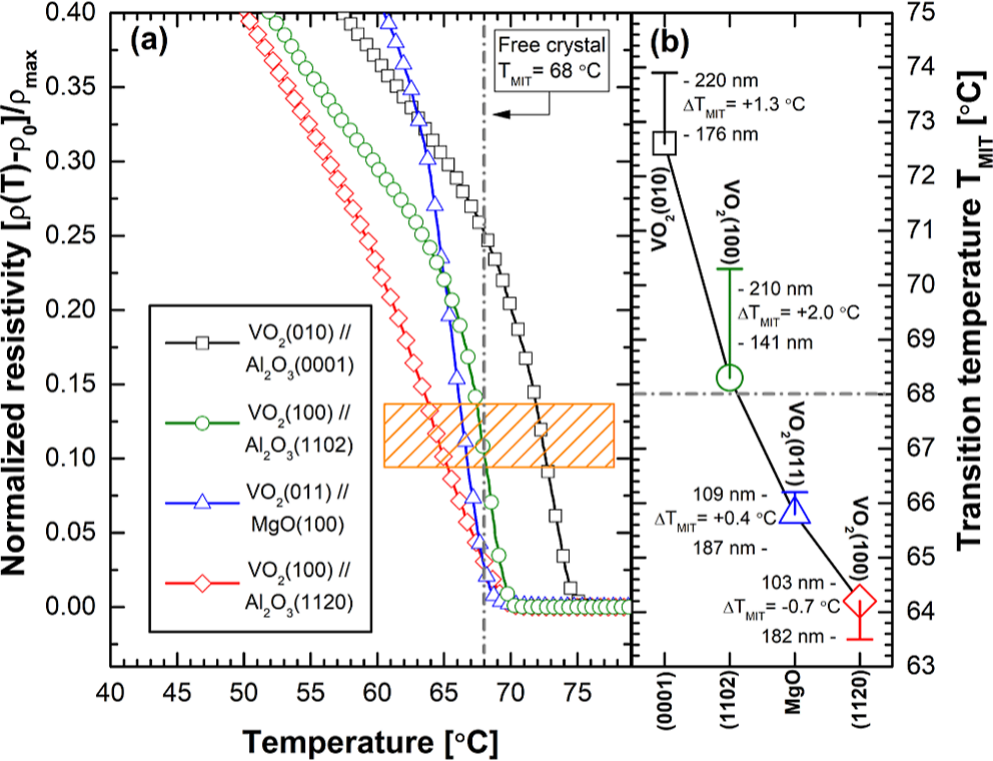
(a) Normalized heating-cycle resistivities as a function of temperature of VO_2_ thin films on various substrates. In the legend, the preferred epitaxial crystalline orientation of the films on different substrates are also shown. (b) Descending values of *T*_MIT_ determined as dρ(*T*)/d*T*_max_, and found in the orange shaded rectangular, of samples in (a) arranged with respect to the type of substrate. Error bars indicate variation of MIT temperature Δ*T*_MIT_ and film thickness *t*_f_ within each group of samples.

In this case, the data of 176 nm thick VO_2_ film with preferred (010) out-of-plane orientation on *c*-cut (0001) Al_2_O_3_ substrate, later denoted also as VO_2_(010)∥Al_2_O_3_(0001), is shown. The point of fastest change in resistivity, i.e., maximum values of dρ(*T*)/d*T*, was used for the determination of MIT temperatures for heating and cooling cycles of the samples. Values of *T*_MIT_ = 72.6 °C and *T*_MIT_ = 66.6 °C were found, respectively, as is also indicated with dashed vertical lines in Figure 1. Later, in this paper, the notation *T*_MIT_ refers only to heating cycle, i.e., to MIT temperature of transition from low temperature insulator phase to high temperature metallic phase. From the inset, the magnitude of ∼3.5 × 10^3^ of MIT in resistivity for VO_2_ on *c*-cut Al_2_O_3_ was found, indicative also of a high-quality epitaxial film structure. In Figure 2a, MIT properties of the other studied VO_2_ films are also shown.

Now the resistivities of the heating cycle are shown in the normalized form [ρ(*T*) – ρ_0_]/ρ_max_, where ρ_0_ is high temperature metallic-phase resistivity and ρ_max_ = ρ(RT). This presentation transforms each ρ(*T*) between the values of 0...1 and thus brings out the mutual order of the *T*_MIT_ by placing the individual values of dρ(*T*)/d*T*_max_ of each sample within the shaded orange rectangle. From the figure legend, also the preferred orientations of the other samples become clear. If values of *T*_MIT_, magnitude of MIT in resistivity (×10^3^), and film thickness *t*_f_ are also presented, the following list can be presented: VO_2_(010)∥Al_2_O_3_(0001) *c*-cut: *T*_MIT_ = 72.6 °C, ∼3.5, 176 nm; VO_2_(100)∥Al_2_O_3_(1102) *r*-cut: *T*_MIT_ = 68.2 °C, ∼4.5, 141 nm; VO_2_(011)∥MgO(100): *T*_MIT_ = 65.8 °C, ∼3.0, 187 nm; VO_2_(100)∥Al_2_O_3_(1120) *a*-cut: *T*_MIT_ = 64.2 °C, ∼4.0, 103 nm, respectively. In Figure 2b, the most interesting property of the samples is brought up by just listing the samples according the descending *T*_MIT_. It was found that difference of the MIT temperatures is as high as Δ*T*_MIT_ = 10.4 °C in maximum between the samples, and it does not seem to correlate with film thickness, preferred orientation, i.e., orientation of the M1 phase *a*-axis, or magnitude of MIT. Error bars and the adjacent labels show the variation of *T*_MIT_ and corresponding film thickness within each type of samples, and it becomes obvious from Figure 2b that MIT temperatures are categorized according to the type of substrate. Transition temperatures of VO_2_ films on *r*- and *c*-cut Al_2_O_3_ were also found higher than *T*_MIT_ = 68 °C of free crystal, dashed lines in Figure 2a,b, whereas the samples on MgO and *a*-cut Al_2_O_3_ were lower, indicative of very complex relationship between the VO_2_ film and substrate. Data presented in Figure 2b also rule out the possible significant oxygen vacancy concentration variation-induced change of *T*_MIT_ between the sample structures studied here, and on the contrary, it actually confirms strong substrate effects.^22^

Hysteresis of MIT, determined as the difference between *T*_MIT_ during the heating and cooling cycles, as pointed out by the dashed vertical lines in Figure 1, for VO_2_ thin-film samples on *a*-cut Al_2_O_3_, MgO(100), *r*-, and *c*-cut Al_2_O_3_ substrates, i.e., in the order of increasing *T*_MIT_, were 3.35 ± 0.85, 5.20 ± 0.2, 6.48 ± 0.49, and 5.45 ± 0.70 °C, respectively. MIT effect resistivity measurements, like data presented in the inset of Figure 1, were actually measured repeatedly several times without any changes in the loops. This implies that consequent transition sequencies M1 → R and R → M1 are identical between the loops, and most likely independent. Hysteresis was found to follow the change of *T*_MIT_ monotonically, but values of hysteresis might also have contribution of temperature change of R → M1 transition upon cooling due to different interface structures. However, this does not seem to have an effect on *T*_MIT_.

In addition to these parameters, one can also notice that steepness of the normalized resistivity change is much higher for the VO_2_ film on MgO(100) in comparison to samples on Al_2_O_3_ substrates. This also actually infers to the very essential thermo-physical substrate effect on MIT properties of VO_2_ films. Thus, the detailed comparative study of the VO_2_ thin-film–substrate interface structures is essential to reveal important phenomena of the fundamental physics of MIT effect in VO_2_ nanostructures.

**Figure 3:**
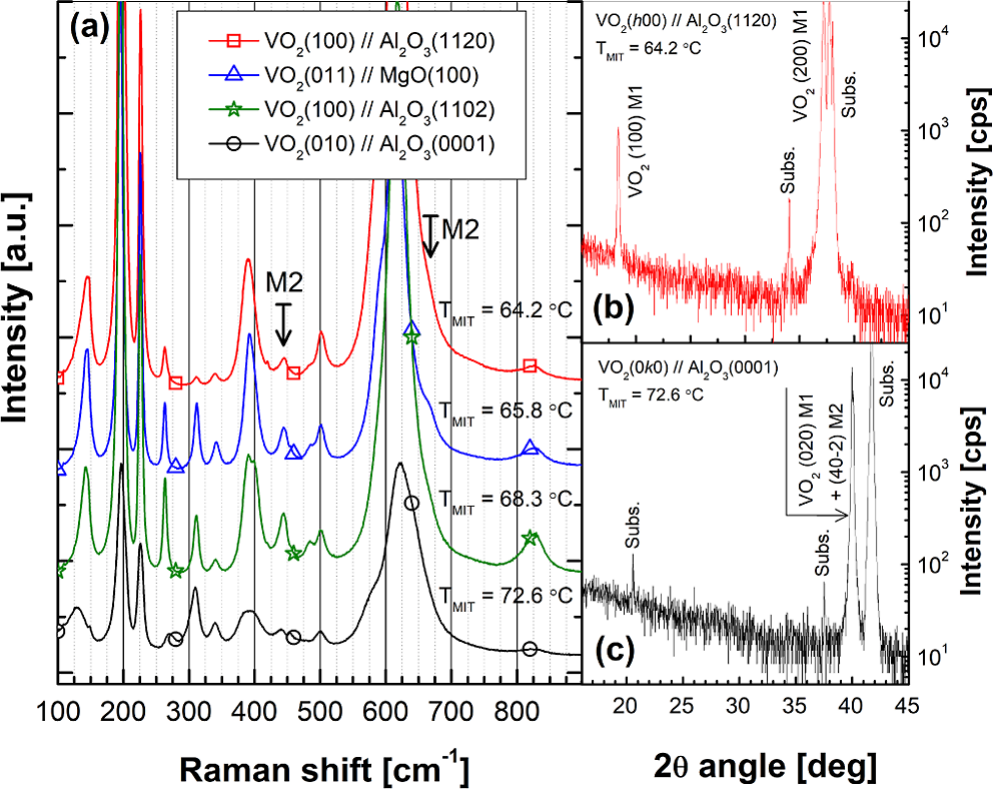
(a) Raman spectra of the four sample structures arranged according to MIT temperature *T*_MIT_. Spectra reveal the polymorphic structure of epitaxial films. Corresponding θ–2θ XRD patterns of (b) VO_2_(100)∥Al_2_O_3_(1120) and (c) VO_2_(010)∥Al_2_O_3_(0001) thin-film–substrate systems. All of the measurements were carried out at the room temperature.

Detailed structural analysis of the VO_2_ samples was carefully performed using Raman spectroscopy, XRD, scanning and tunneling electron microscopy (SEM and TEM), and XPS methods. In addition to confirming the phase structure, crystalline quality, and high epitaxial orientation of the thin films, these methods can be used to reveal the nanometer and atomic-scale microstructural details and compositional stoichiometry also in the interface between VO_2_ thin film and substrate. Raman spectra, recorded at room temperature, of the four different sample type are shown in Figure 3a. It can be clearly seen that all nine A_g_ and nine B_g_ Raman modes predicted by the group theory for *P*2_1_/*c* (M1) symmetry of monoclinic low-temperature phase of VO_2_ are seen.^23^ Especially, considering the peaks around ∼140, ∼200, ∼225, ∼390, and ∼620 cm^–1^, very high intensity and narrow Raman modes are found, indicative of a congruent and uniform phase structure. Preferred crystalline orientation with respect to laser-light polarization in the surface plane of thin-film also contributes to the mutual intensity ratio of the specific modes between the samples. This is seen by comparing Raman spectra of samples VO_2_(100)∥Al_2_O_3_(1120) and VO_2_(010)∥Al_2_O_3_(0001) in Figure 3a, at around wavevector ∼310 cm^–1^, for example, where the (010) oriented sample clearly amplifies A_g_ modes. In addition, the splitting of ∼390 cm^–1^ mode is solely due to orientation effects. However, in more rigorous analysis, Raman spectra were found to have features indicative of possible presence of high-temperature monoclinic *C*2/*m* (M2) insulator phase, as well.

If one should consider VO_2_ thin films consisting of only pure monoclinic *C*2/*m* (M2) insulator phase, the Raman modes around ∼450 and ∼660 cm^–1^, pointed out also by the arrows in Figure 3a, should be the dominant peaks.^24,25^ The mode at ∼445 cm^–1^ appears in both M1 and M2 phases, but the higher wavenumber shoulder ∼455 cm^–1^ belongs only to M2, and is here seen in three samples VO_2_(011)∥MgO(100), VO_2_(100)∥Al_2_O_3_(1102), and VO_2_(010)∥Al_2_O_3_(0001). Similarly, the contribution of the ∼660 cm^–1^ mode is only found in these samples. Spreading of the Raman mode around ∼310 cm^–1^ toward lower wavenumbers, as seen in sample VO_2_(010)∥Al_2_O_3_(0001), can be also interpreted as an indication of the existence of the M2 phase, which is known to emphasize the splitting of degenerate A_g_ and B_g_ modes. Finally, the Raman mode at around ∼610 cm^–1^, belonging only to the M1 phase, of the sample VO_2_(010)∥Al_2_O_3_(0001) has clearly higher full width half-maximum (FWHM) value in comparison to other samples. This is true even if we consider the possible effects of M2 phase mode ∼660 cm^–1^, and *c*-cut Al_2_O_3_ substrate Raman mode ∼575 cm^–1^. As shown by Tselev et al., VO_2_ is basically a ferroelastic material, and Ginzburg–Landau theory can be thus applied to predict its possible phase transitions.^26^ This led to Raman spectroscopy observation of highly distorted metastable M1 phase, possibly with the triclinic symmetry, also spreading the ∼660 cm^–1^ mode of VO_2_. Later in this paper, we show TEM micrographs proving the existence of such, only a few unit cells thick, layers in the interface of the VO_2_(010)∥Al_2_O_3_(0001) system, which we assume to be the highly distorted M1 phase with a symmetry close to VO_2_ (*P*4_2_/*nmc*) A phase.^27,28^

**Figure 4:**
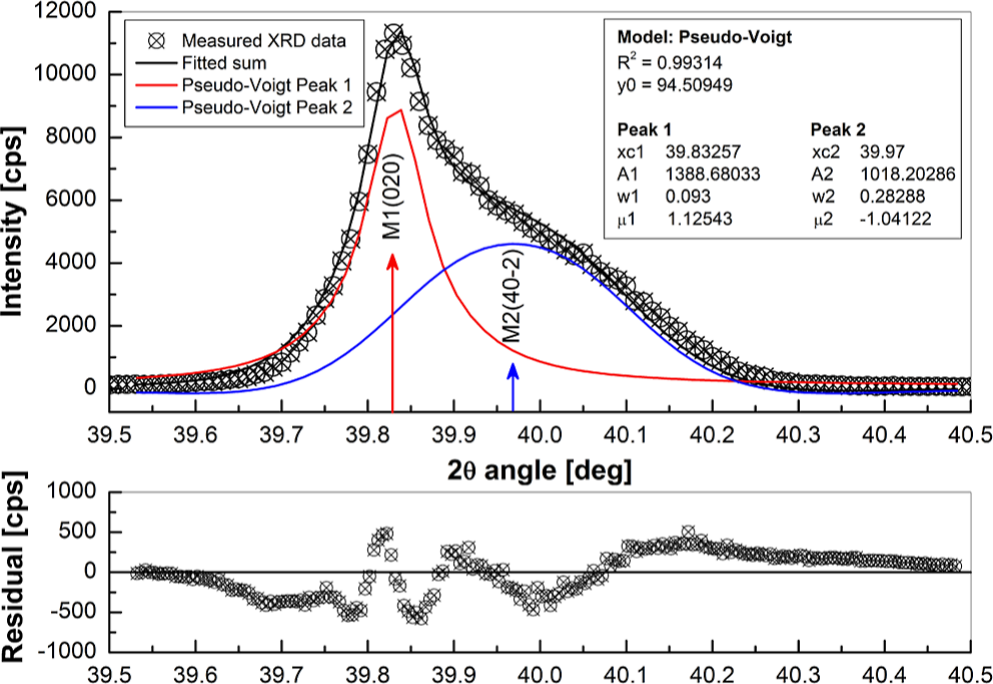
High-resolution XRD pattern measured between 2θ angles from 39.5 to 40.5°, and fitted with two Pseudo-Voigt functions, of the sample VO_2_(010)∥Al_2_O_3_(0001). The legend shows the fitting parameters, and the fitting residual is seen in the lower graph.

XRD patterns with θ–2θ scans measured of the samples VO_2_(100)∥Al_2_O_3_(1120) and VO_2_(010)∥Al_2_O_3_(0001) are shown in Figure 3b,c, respectively. Both films show very sharp and high intensity reflections from single lattice plane groups, namely, (*h*00) for VO_2_ thin film on *a*-cut, and (0*k*0) on *c*-cut Al_2_O_3_. No other M1-phase reflections were found, indicative of epitaxial major M1-phase VO_2_ thin-film structures with a strong preferred orientation. Even though it would be very appealing to label reflection at 2θ ≈ 20.5° in sample VO_2_(010)∥Al_2_O_3_(0001) in Figure 3c as the (010) reflection of the M1 phase, the structure factor makes (0, 2*n* + 1, 0) reflections forbidden for *P*2_1_/*c* symmetry.^29^ This XRD peak can also originate from the *c*-cut Al_2_O_3_(0001) substrate, as it is now labeled. However, one should bear in mind that, if the M1 phase would be very strongly distorted, for example, due to strain, structure factor would give up, and small reflection could be seen in the XRD pattern, exactly in the same way what is seen in the case of single crystalline silicon wafers measured with high accuracy 2θ-angle positioning. The possible existence of the *C*2/*m* (M2) insulator phase was separately studied with high-resolution XRD measurements, as shown for the VO_2_(010)∥Al_2_O_3_(0001) sample in Figure 4.

Here, reflection between 2θ angles from 39.5 to 40.5°, labeled as VO_2_ (020)M1 + (40–2)M2 also in Figure 3c, was measured using only Cu Kα_1_ radiation, i.e., Cu Kα_2_ and Cu Kβ were stripped of from the X-ray beam, with a very slow scanning speed, also at the room temperature. Two Pseudo-Voigt functions with 2θ angle positions ∼39.83 and ∼39.97° were fitted to intensity data after a linear background removal, and a reasonable fitting, with *R*^2^ ≈ 0.993 and residual intensity shown in lower graph was achieved. Reflection at 2θ ≈ 39.83° originates evidently from (020) planes of monoclinic M1 phase, whereas the peak at 2θ ≈ 39.97° does not and can actually be designated to monoclinic M2-phase reflection from (40–2) planes. This finding resembles very closely to what was found by Okimura et al.^30^

**Figure 5:**
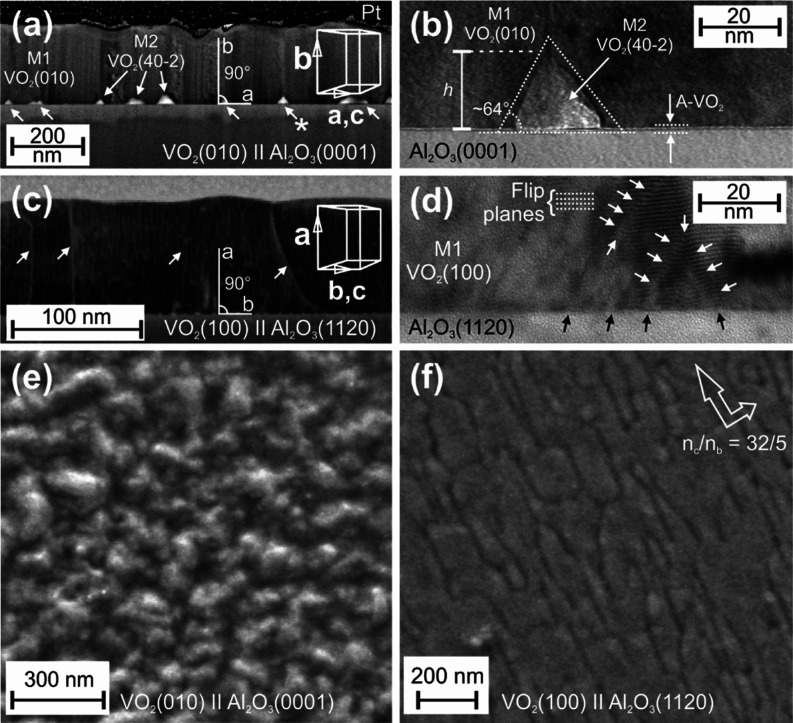
Four cross-section TEM micrographs of (a,b) sample VO_2_(010)∥Al_2_O_3_(0001) and of (c,d) sample VO_2_(100)∥Al_2_O_3_(1120). M2-phase cones in (a,b) and TD lines in (c,d) are marked with various arrows. Corresponding VO_2_ thin-film surface SEM micrographs are shown in (e,f), respectively.

Results presented in Figures 3 and 4 suggest that, despite the fact that the majority phase of the VO_2_ thin films on *a*-, *r*-, *c*-cut Al_2_O_3_, and MgO substrates is highly oriented epitaxial monoclinic M1 phase, there are clear indications of strongly distorted M1 phase, close to some other lower symmetry, and monoclinic M2 phase as well, also present in the samples at room temperature. Generally, this is believed to occur as an adaptation process to elastic misfit strain ε in the thin-film–substrate interface, as discussed above. In Figure 5, there are four cross-section TEM micrographs of sample VO_2_(010)∥Al_2_O_3_(0001) in (a) and (b), and of sample VO_2_(100)∥Al_2_O_3_(1120) in (c) and (d).

From the conventional phase diagram of a free VO_2_ crystal it is well known, as also presented Figure 9a, that a free crystal contains only the M1 phase, and the M2 phase exists only under strain. While deposited, films are initially growing in the tetragonal R-phase, and strain state leads to construction containing M1 or M2, or coexistence of those phases during the cooling. In a thin-film–substrate system, misfit strain originates from the film–substrate interface, and is relaxed in the film thickness direction. Thus, M2 phase is to be found in the interface, where strain state has maximum. Intensity ratios of both XRD and Raman spectra clearly reveal that M2 is the minority phase, as can be also concluded from Figure 5.

Corresponding VO_2_ thin-film surface SEM micrographs are shown in Figure 5e,f, respectively. All cross-section micrographs in Figure 5a–d confirm congruent and uniform high-quality epitaxial VO_2_ thin films of M1 as a major phase without any grain boundaries. However, in the film–substrate interface of sample VO_2_(010)∥Al_2_O_3_(0001), the presence of M2 phase of VO_2_ is clearly seen in the form of pyramid-shaped cones, as pointed out by the white arrows in Figure 5a.^31^ These M2-phase cones are also found to be brighter in comparison of the other parts of the micrograph indicative of their higher electrical conductivity. Presented unit-cell coordination drawings with lattice constants (*a*, *b*, and *c*) are also confirmed by both XRD data and TEM micrograph. In Figure 5b, there is a magnification of one M2-phase cone pointed out by the white arrow with asterisk in Figure 5a. The height *h* of the cones was typically below ∼25 nm, and the angle between the cone wall and substrate surface was ∼60–70°. As the misfit strain energy *E*_ε_, inducing M1 → M2 phase transition of VO_2_ thin film in the interface, decreases as a function of increasing distance in the direction of film thickness, the volume ratio of M2 phase decreases consequently. Since the M2 phase is also epitaxial with (40–2) planes parallel to the film–substrate interface, the angle of the interface between cone-shaped M2 and M1 phases follows the corresponding M2 phase crystal plane (*hkl*), minimizing the strain energy. Finally, the strain minimizes to the level not capable to induce M1 → M2 phase transition anymore, and a TD is generated. More importantly, Figure 5b reveals another anomaly in the sample VO_2_(010)∥Al_2_O_3_(0001) interface. A uniform layer of 2–3 unit cells, maximum ∼15 Å in thickness, was observed clearly in the interface, as pointed out by two horizontal dashed lines, and labeled as the A-VO_2_ phase. The new layer is assumed to be just another phase transformation, or distortion, of the M1 phase due to relaxation of high initial tensile strain in the interface, converting the mechanical strain energy in to the form of chemical enthalpy of the distorted structure. Finding supports perfectly the observed Raman spectroscopy and XRD results in Figure 3, and the subsequent discussion of possible existence of highly distorted M1 phase with a symmetry close to the VO_2_ (*P*4_2_/*nmc*) A phase in the films.^27,28^ At this point, it is very important to bring up the so-called mask-free patterned sapphire substrate (PSS) technology. In the PSS technique, the sapphire substrate surface is patterned exactly with a similar pyramid-shaped cones as found in the VO_2_(010)∥Al_2_O_3_(0001) interface in the form of M2 phase of VO_2_, by using, for example, reactive ion etching. This is done in order to annihilate the TDs otherwise appearing in very high numbers due to high tensile misfit strain in the interface between the sapphire substrate and GaN heteroepitaxial films.^32−34^ Values of TDD ≈ 4.7 × 10^7^ cm^–2^ were reported ensuring the high performance of GaN structures in LED technology, for example. From this, it can be concluded that M2-phase cones of VO_2_ also found in the VO_2_(010)∥Al_2_O_3_(0001) interface have their origin in thickness-dependent strain energy relaxation through the conversion in to the chemical enthalpy of polymorphic M2 phase. This holds also for the very thin layer of the VO_2_ A phase.

Existence of this interface layer with the thickness of few unit cell, in addition to M1 and M2 phases, in VO_2_(010)∥Al_2_O_3_(0001) films was confirmed by TEM studies. Although, it was not possible to deduce the exact crystal symmetry of such a small entity, XPS measurements hinted that this layer would be stoichiometric VO_2_. Thus, it was assumed that layer is highly disordered M1 phase with symmetry close to the VO_2_ A phase. Since films grow epitaxially in the R phase at elevated temperatures, the M1 phase is transformed directly on *c*-Al_2_O_3_ surface upon cooling, and it leads to unrealistic high strains. Existence of this layer in thus an adaptation to this transition. As it is shown in detail in several studies, the other candidate for this layer would be the tricilinic phase T. However, the A phase has higher symmetry than T.^26−28^

Moving on to Figure 5c,d, there are cross-section TEM micrographs of the sample VO_2_(100)∥Al_2_O_3_(1120) interface shown. It becomes obvious that pyramid-shaped cones of M2 phase of VO_2_, as well as A phase, are now totally missing, and the substrate–film interface was found very sharp. Since the initial strain of M1 phase in *a*-axis lattice direction, which is actually the thermodynamic order parameter for polymorphic phase transition from M1 to M2 phase, on *a*-cut sapphire, is compressive, i.e., ε_0_ < 0, the adaptation to high misfit strain through transitions into the polymorphic VO_2_ phase with a larger unit cell volume is not allowed. This actually leaves the dislocations as only mechanism for strain relaxation, which are also clearly seen as TD lines, pointed out by the white arrows, in Figure 5c,d.^35^ Theoretical initial strain  was extremely high leading to critical thickness values in the scale of Burger vector, and thus dislocations are nucleated already in the film–substrate interface during the phase transitions even in the thinnest films with *t*_f_ ≈ 100 nm, as expressed by the black arrows in Figure 5d.^18,21^ As the chemical composition of vanadium dioxide VO_2_, as well as, of the other vanadium oxide compounds, is hardly ever stoichiometric, but rather more correctly expressed as VO_2–*x*_, it has the tendency to compensate the spatial valence charge difference by forming twinning flip plane structures, as can be also seen in Figure 5d with higher magnification.^27,36^ Finally, SEM micrographs of VO_2_ thin-film surfaces on *c*-cut and *a*-cut Al_2_O_3_ substrates are shown in Figure 5e,f, respectively. As both of the surfaces show optical flatness quality with rms surface roughness values *R*_q_ < 5 nm, the sample VO_2_(100)∥Al_2_O_3_(1120) surface is actually atomically flat with *R*_q_ < 1 nm, if the oriented TD pattern is neglected. The TD pattern was also found to be oriented according to the VO_2_ M1 phase unit-cell basal plane (0*kl*) direction, as shown by unit-cell coordination drawing in Figure 5c. After Fourier analysis, the ratio of periodicities of *n*_c_/*n*_b_ = 32/5 was found, as also shown in Figure 5f.

**Figure 6:**
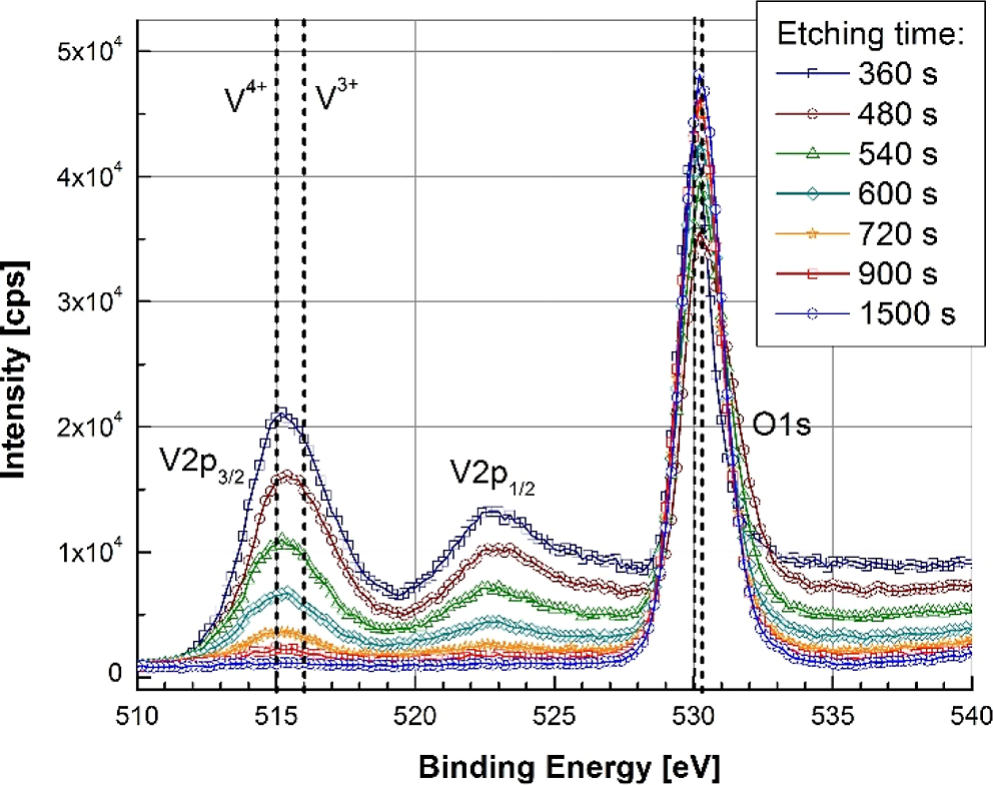
XPS spectra measured through a VO_2_(010)∥Al_2_O_3_(0001) film thickness after various etching times. Main intensity peaks around vanadium V 2p_3/2_, V 2p_1/2_, and O 1s are pointed out.

Samples of this study were selected from numerous samples fabricated by the PLD process with various parameter sets, such as laser pulse properties, temperature, and partial oxygen pressure, leading in to different microstructures including amorphous, nanocrystalline, columnar, and epitaxial films. Only epitaxial films, confirmed by XRD, SEM, and TEM data were selected to this study. An example confirming the epitaxy, flip-planes of VO_2_(100)∥Al_2_O_3_(1120), also shown in Figure 5d, are composed of sets of same crystal lattice planes clearly reaching over the distances longer than film thickness. In addition, composition ratio of an epitaxial film VO_2_(010)∥Al_2_O_3_(0001), as a function of film thickness *t*_f_ is presented in Figure 6. Composition was found to stay unchanged close to the VO_2_ bulk value.

XPS measurements confirmed the chemical stoichiometry of the VO_2_(010)∥Al_2_O_3_(0001) sample as a function of film thickness *t*_f_ starting from the free surface. XPS spectra showed two main intensity peaks around vanadium V 2p_3/2_ and V 2p_1/2_ levels with energies of 515.25 and 522.75 eV, respectively. It is important to notice that spectra maxima, as well as the shape of the intensity peaks, maintained unchanged throughout the film to the point of disappearance when pure substrate was revealed. This is an indication of constant composition distribution with V^4+^ valence as main vanadium bond, and thus, XPS results do not support the idea of existence of other phases than VO_2_ polymorphs, such oxidation states as V_2_O_3_ or V_2_O_5_, even in the vicinity of film–substrate interface.

The observed anomalies and deviations from major M1 phase of VO_2_ thin-film structures are characteristic for the low-temperature insulating state at room temperature. Clearly lower values of room temperature electrical resistivity ρ(RT = 25 °C) of the insulator state of VO_2_ thin films on *c*-Al_2_O_3_ substrates also suggests that the interface layer, consisting mainly of M2 and A phase, have ∼3 times higher conductivity in comparison to main M1 phase of the film. When the effect of film thickness *t*_f_ variation, in this case *t*_f_ = 105–220 nm, on the electrical resistivity in terms of surface scattering, i.e., Fuchs–Sondheimer effect, is also considered, the change in resistivity should be much smaller, in the range of Δρ < 0.01. Together with Raman spectroscopy, XRD, and TEM results, this deviation from the conventional Fuchs–Sondheimer model, suggests the formation of specific layer in the VO_2_(010)∥Al_2_O_3_(0001) interface with the conductivity properties typical to the 2DEG topological insulator reconstruction layer, as a result of VO_2_ film adaptation to high misfit strain at the equilibrium state at room temperature.^37^ Since the M1 phase of the VO_2_ film has electrical conductivity close to that of slightly doped n-type Si, and *c*-Al_2_O_3_ substrate represents extreme insulator at room temperature, in turn, one can hardly apply the theory of polar catastrophe induced charge discontinuity compensation for the origin of interface reconstruction in the VO_2_(010)∥Al_2_O_3_(0001) system. However, polymorphic phase transitions and related symmetry distortions, defects and, in the extreme case, possible amorphization, and even the piezoresistive effect due to residual mechanical strain, definitely induce spatial charge distribution variations and energy band shifts leading to the observed changes of the room-temperature electrical conductivity in the thin film-interface layer.

## 4 Modeling of Interface Strain State

**Figure 7:**
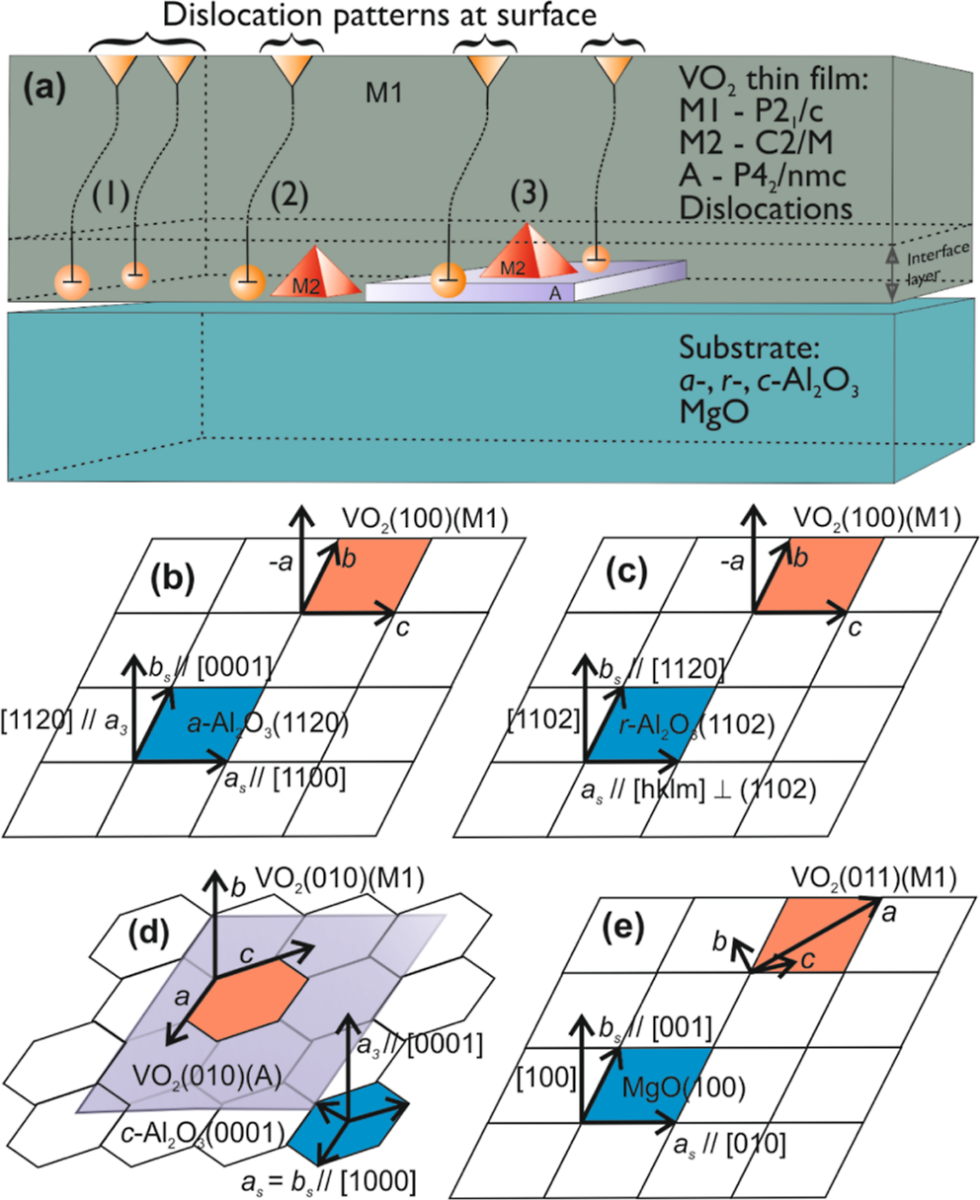
(a) Schematic drawing of the mechanisms (1) D, (2) D + M2, and (3) D + M2 + A appearing on the VO_2_ thin-film side in the vicinity of the film–substrate interface forming a kind of critical disordered interface layer with the thickness of few tenths of nanometers in maximum. Four schematic drawings describing the interface structure and setting the coordinate systems for strain-state theoretical modeling (b) for VO_2_(100)∥Al_2_O_3_(1120), (c) for VO_2_(100)∥Al_2_O_3_(1102), (d) for VO_2_(010)∥Al_2_O_3_(0001), and (e) for VO_2_(011)∥MgO(100) structures.

Collecting together the main experimental results presented above, it is possible to conclude that there are three different mechanisms through which the interface lattice misfit induced mechanical strain energy *E*_ε_ is converted and relaxed in the phase transition from the high-temperature tetragonal *P*4_2_/*mnm* (R) metal phase to the low-temperature monoclinic *P*2_1_/*c* (M1) insulator phase on event of the MIT effect. These mechanisms include formation of dislocations (hereafter marked as D), emergency of monoclinic *C*2/*m* (M2) polymorphic phase, and emergency of, most likely, another polymorphic phase with the symmetry close to the tetragonal *P*4_2_/*nmc* (A) phase. All these mechanisms appear on VO_2_ thin-film side in the vicinity of the film–substrate interface forming a kind of critical disordered interface layer with the thickness of few tenths of nanometers in maximum, as is also described in schematic drawing in Figure 7a. Disordered interface layer actually ensures the existence of a highly oriented congruent epitaxial M1-phase VO_2_ thin film by absorbing the misfit strain energy through relaxation and phase transitions, and certainly it has effects on the reversibility properties of MIT effect. As all of the mechanisms (D, M2, and A) are also intermediate metastable deviations of, and inside, the stable room-temperature M1 phase, they can be seen as obstacles for the phase transition M1 → R due to their formation enthalpy and thus have an impact on the value of *T*_MIT_ and hysteresis behavior of the MIT effect.

In Figure 7a, there are three scenarios presented for the disordered interface layer, namely, (1) for VO_2_(100)∥Al_2_O_3_(1120) *a*-cut with *T*_MIT_ = 64.2 °C and mechanism D, (2) for VO_2_(100)∥Al_2_O_3_(1102) *r*-cut with *T*_MIT_ = 68.2 °C (and VO_2_(011)∥MgO(100) with *T*_MIT_ = 65.8 °C) and mechanisms D + M2, and (3) for VO_2_(010)∥Al_2_O_3_(0001) *c*-cut with *T*_MIT_ = 72.6 °C and mechanisms D + M2 + A. These scenarios are obvious conclusions from the experimental results, and furthermore, it is important to notice, that the measured value of *T*_MIT_ increases monotonically with the increase in complexity of the disordered interface with mechanisms D, D + M2, and D + M2 + A, respectively, in different VO_2_ film–substrate systems. In addition, the hysteresis is clearly higher for the films with the mechanisms M2 and M2 + A.

In Figure 7b–e, there are four schematic drawings presented, describing the interface structure and setting the coordinate systems for strain-state theoretical modeling for samples VO_2_(100)∥Al_2_O_3_(1120), VO_2_(100)∥Al_2_O_3_(1102), VO_2_(010)∥Al_2_O_3_(0001), and VO_2_(011)∥MgO(100), respectively. A two-dimensional interface is presented as a rectangular mesh (*x*, *y*), which is not in scale, and is actually hexagonal in the case of the *c*-cut Al_2_O_3_ substrate in Figure 7d, on which the VO_2_ thin-film M1-phase orientation (*hkl*) and unit-cell axis (*a*, *b*, *c*) are defined by coordinate axis with orange, and for substrate surface orientation (*hkl*) and unit-cell axis (*a*_s_, *b*_s_, *c*_s_) with blue, basal plane. Direction perpendicular to interface mesh, i.e., in the direction of film thickness is assigned also as *z*-axis. Also, the highly distorted M1 phase with a symmetry close to VO_2_ (*P*4_2_/*nmc*) A phase, which existence in the VO_2_(010)∥Al_2_O_3_(0001) interface system was proven by the XRD, Raman spectroscopy, and TEM results presented in Figures 3 and 5, respectively, is depicted in Figure 7d by the violet rectangular. In the case of *a*-cut and *r*-cut Al_2_O_3_, and MgO substrates VO_2_ thin-film lattices were fitted directly on the substrate surface plane, where as in the case of *c*-cut Al_2_O_3_ substrate VO_2_(010) A-phase lattice plane was used as for fitting. This assumption was done due to fact that A phase is clearly the closet polymorphic symmetry minimizing the strain state and maintaining the chemical stoichiometry, as required by XPS results. VO_2_ thin-film M1-phase orientations (*hkl*) were determined by using XRD measurements and initial strain-state analysis, as described previously.

It is essential to express here, that MIT effect of a single-crystalline bulk VO_2_ exhibits a thermoelastic phase transition between two phases, namely, the metallic high temperature phase of rutile structure, i.e., R phase with tetragonal *P*4_2_/*mnm* symmetry, and the insulating low temperature phase, i.e., M1 phase with monoclinic *P*2_1_/*c* symmetry. This transition is considered to be an abrupt and diffusionless first-order phase change between the two crystalline structures, and the fundamental equation of Gibbs free energy *G* balancing the transformation under the applied external mechanical force *F* can be written as
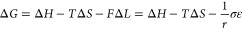
1where Δ*H* is the latent heat and Δ*S* is the entropy change of the transition, Δ*L* is the dilation due to transition in the direction of force *F*, and ε and σ are the corresponding linear strain and the uniaxial stress, respectively. *r* is the density of material. Under the constant steady-state conditions, differentiation of eq 1 at Δ*G* = 0 leads to the famous Clausius–Clapeyron relation of modified transition temperature *T* with respect to uniaxial stress σ
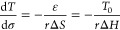
2where *T*_0_ is now the transition temperature *T*_MIT_ of a free crystal.^38^ Negative sign in eq 2 stands now for the uniaxial compressive strain and stress, as it is the case for hydrostatic pressure, for example. For single-crystalline bulk VO_2_, the relationship describes a linear relationship between the stress and the temperature and makes it possible to draw a simple phase diagram.^39^ However, in the case of constrained nanostructured VO_2_ thin films with structural deviations through the mechanisms D, D + M2, and D + M2 + A, the linear relationship is hardly valid anymore, and therefore, the concept of phase co-existence in the thin-film structures actually becomes necessary to explain the relationship and to draw the correct phase diagram, as was also pointed out by Park et al.^40^

Strain-state analysis of thin films on substrates is similar to general plane stress conditions due to two-dimensional nature of the problem. In the usual notation, the film is loaded in the interface plane (*x*, *y*), and the zero stress components are σ_*z*_ = τ_*yz*_ = τ_*zx*_ = 0, where τ stands for shear stress. Thus, three-dimensional isotropic linear elastic stress–strain relation {σ} = [*Y*]{ε} takes the following simple two-dimensional matrix form
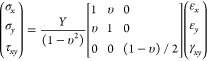
3where *Y* is Young’s modulus, υ is the Poisson ratio, and γ_*xy*_ is the shear strain. It should be also noted here that even though the stress component σ_*z*_ = 0, the strain component ε_*z*_, i.e., strain in the direction of film thickness, is nonzero and can be calculated with equation^41^
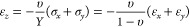
4

The definition of strain ε_*i*_ itself is based on an initial state, length *L*_0_, against which the final state *L*_1_ is compared. Thus, the strain, i.e., Cauchy strain or engineering strain, is expressed as the ratio of the change in length Δ*L* per unit of the original length *L*_0_ of a material line element so that ε = Δ*L*/*L*_0_ = (*L*_1_ – *L*_0_)/*L*_0_. When applied in to the present thin-film–substrate interface system, comparison is made between substrate lattice constant *a*_s_, which compares to static final state *L*_1_, and the corresponding thin-film lattice constant *a*_f_, so that ε = (*a*_s_ – *a*_f_)/*a*_f_, which is also called as misfit strain. As was already pointed out, in the Mott theory of metal insulator transition, the strain along the VO_2_ M1 phase *a*-axis, i.e., ε_*a*M1_, is the most critical parameter controlling the phenomenon. Thus, strain ε_*a*M1_ is called as a thermodynamic order parameter, and as it refers to direct electro–electron correlation induced pure Mott transition and according to eqs 1 and 2 it holds exceptionally well for VO_2_ single crystals and, in the limited parameter area, for nanobeams, but fails dramatically often in the case of thin films.^39,40^ From the elasticity and mechanical strain energy *E*_ε_ point of view, pure dilation by ε_*a*M1_ of VO_2_ unit cell lattice refers to energy related to volume change *E*_vol_, and is thus indication of pure Mott transition. In comparison, the strain energy inducing the distortion of the symmetry *E*_dist_, so that *E*_ε_ = *E*_vol_ + *E*_dist_, refers to distortion of the symmetry element, and can be considered as an indication of SPT process through Peierls mechanism. However, also here, ε_*a*M1_ is considered carefully in both experimental and theoretical context, and its effects on *T*_MIT_ and other properties are studied in detail.

Calculation of the initial strain of the studied structures with the VO_2_ M1 phase at room temperature leads to values |ε_0_| > 2.5% at least in one lattice direction in the in-plane interface, and thus, maintaining the highly oriented epitaxial film structure upon the cooling from high temperature R phase would hardly be possible without cracking or amorphization of the structure. First mechanism to decrease the misfit Cauchy strain is the formation of dislocations D on the VO_2_ film side of the interface, whenever the strain is tensile in any direction *i* in the interface plane (*x*, *y*). In formation of dislocation D, there are (*n*_*i*_ + 1) unit cells of VO_2_ thin film M1 phase λ_f_^M1^ = (*a*_M1_, *b*_M1_, *b*_M1_) stretched over the *n*_*i*_ unit cells of substrate lattice constant . With appropriate selection of the adjacent lattice parameters across the interface plane from matrices λ_f_^M1^ and , the misfit Cauchy strain relaxed by the dislocation mechanism D can be simply expressed now as ε_*i*_ = [*n*_*i*_λ_s*i*_ – (*n*_*i*_ + 1) λ_f*i*_^M1^]/[(*n*_*i*_ + 1) λ_f*i*_^M1^], where *n*_*i*_ is called as a dislocation parameter in the direction *i*. The second mechanism to decrease the misfit Cauchy strain is the formation of polymorph phases of the VO_2_ thin film, such as M2, A, or even R phase, in the film–substrate interface. Since the M1 phase is still the initial state, the adjacent unit-cell lattice constant of VO_2_ film stretched over the unit cells of substrate lattice constant  is now changed by the difference induced by one transformed element of polymorph phase lattice constant matrix λ_f_^P^ = (*a*_P_, *b*_P_, *c*_P_), i.e., *T*_*i*_λ_f*i*_^P^, in to the lattice constant of original M1 phase, so that λ_f*i*_^M1^ → [(*T*_*i*_λ_f*i*_^P^ – λ_f*i*_^M1^) + λ_f*i*_^M1^]. For example, in the case of polymorphic transition from (M1) *P*2_1_/*c* symmetry to (M2) *C*2/*m* symmetry, as happens in the strain relaxation mechanism D + M2, the lattice symmetry operation is well known, and can be expressed as matrix representation λ_f_^M1^ = *T*·λ_f_^P^ as follows^26^
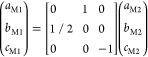
5

Above analysis sounds trivial, but is of crucial importance in order to calculate the final and relaxed direction of the strain correctly in comparison to the free M1 phase as the reference state, not to new M2 phase, and from eq 5 it can be seen that *b*_M1_ = 1/2*a*_M2_, and thus . Consequently, the modified misfit Cauchy strain due to mechanism D + M2 reduces now in to more general form
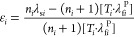
6

Using above formalism, it is now possible to calculate, with the aid of eq 4, all of the misfit Cauchy strain components (ε_*x*_, ε_*y*_, and ε_*z*_), which actually explicitly describe the residual strain state of VO_2_ thin films on various substrates at room temperature. In a more general approach, the term [*T*_*i*_λ_f*i*_^P^] could be considered as a symmetry distortion term due to the misfit strain. Since the strain is found to be three dimensional, even though two-dimensional assumption of interface stress state, i.e., σ_*z*_ = 0, for the steady state is considered, the component ε_*z*_ ≠ 0, and the most important parameter to calculate in such a system is the total residual strain energy^41^
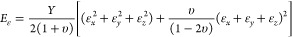
7

In eq 7, the total residual strain energy *E*_ε_ was written omitting the shear effect, thus based on the definition of the misfit Cauchy strain. In phase transition M1 → M2 considered here, there is no shear deformation as defined by eq 5. Obviously, the observed strain relaxation mechanisms D, D + M2, and D + M2 + A actually work to minimize *E*_ε_ instead of necessarily strain ε_*a*M1_, thermodynamic order parameter of single crystalline VO_2_. At this point, it is worth mentioning that in the studied structures in VO_2_(100)∥Al_2_O_3_(1120) ε_*a*M1_ = ε_*z*_ < 0, in VO_2_(100)∥Al_2_O_3_(1102) ε_*a*M1_ = ε_*z*_ > 0, in VO_2_(010)∥Al_2_O_3_(0001)ε_*a*M1_ = ε_*x*_ > 0, and in VO_2_(011)∥MgO(100) ε_*a*M1_ = ε_*x*,*y*_ > 0, respectively. It is also essential to notice that strain energy *E*_ε_ is not necessarily minimized only by the 2D interface-plane strain components ε_*x*_ and ε_*y*_ but also the term  including the out-of-plane strain component ε_*z*_. Thus, it might be profitable from the system energy point of view even to change the sign of the initial strain ε_*i*_ in relaxation process in order to minimize total residual strain energy *E*_ε_.^41,42^ In order to find out the interface configuration and structure leading to minimum residual strain state and energy for each VO_2_ thin film–substrate interface combination, the strain equations using modified Cauchy strains ε_*i*_ according the eq 6, with eq 4, are formulated for each system, as a conclusion of experimental data shown previously, equations are inserted in eq 7, and finding the minimum ∂*E*_ε_/∂*n*_*i*_ = 0 leads to dislocation parameter values *n*_*i*_ = (*n*_*a*_, *n*_*b*_, *n*_*c*_) in each possible VO_2_ thin film–substrate interface directions minimizing the interface misfit residual strain energy. Here, subscripts (*a*, *b*, *c*) refer to VO_2_ M1 phase basal lattice constants in the (*x*, *y*) plane, as shown in Figure 6b–e.

**Figure 8:**
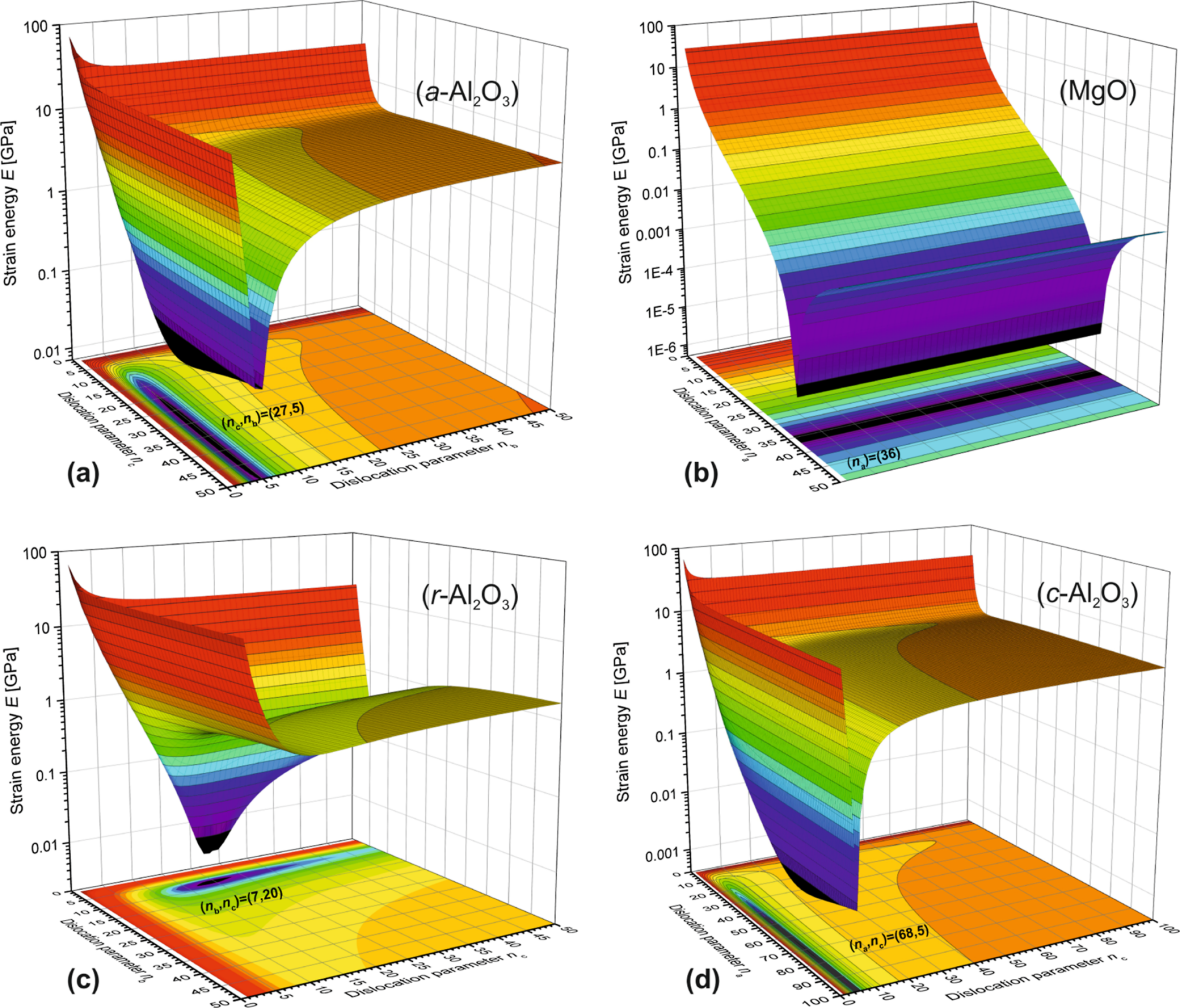
Calculated residual misfit strain energy *E*_ε_ potentials as a function of dislocation and polymorph phase transition effects with parameter *n*_*i*_ for VO_2_ thin films on (a) *a*-cut Al_2_O_3_, (b) MgO, (c) *r*-cut Al_2_O_3_, and (d) *c*-cut Al_2_O_3_ substrates. Exact positions of minima are pointed out by the notations *n*_*i*_ = (*n*_*a*_, *n*_*b*_, *n*_*c*_).

Material parameters, such as Young’s modulus *Y* = 140 GPa and Poisson’s ratio υ = 0.367 for the VO_2_ M1 phase, as well as the lattice constant values of different VO_2_ phases and substrates can be easily found in numerous literature sources and were used finding numerical solutions. In Figure 8, there are four strain energy potentials *E*_ε_ as a function of dislocation parameters *n*_*i*_ shown for the VO_2_ thin-film–substrate interface systems studied here. In general it can be noticed, that strain energy potential minima are explicitly narrow and deep, and especially strongly anisotropic with respect to unit cell axis directions in the interface. As the low-temperature M1 phase of VO_2_ is known to have elastically isotropic mechanical properties, the high-temperature metallic R phase, in turn, shows extensive elastic anisotropy.^43^

The observed anisotropic strain energy potential minima in Figure 8 can thus be understood to promote SPT s away from M1 toward lower symmetries of M2 and A phases, as was also confirmed by the experimental results. In the case of the *a*-cut Al_2_O_3_ substrate shown in Figure 8a, one recalls that initially ε_*a*M1_ = ε_*z*_ < 0, which does not allow the transition from M1 to M2 phase with larger unit-cell volume, thus leaving only mechanism D to relax the misfit strain. Actually, theory leads to a very good correspondence between the calculated values of *n*_*i*_ and the surface morphology features of TD patterns in the VO_2_(100)∥Al_2_O_3_(1120) system, for example, where (*n*_*b*_, *n*_*c*_) = (5, 27). The theoretically calculated ratio *n*_*c*_/*n*_*b*_ = 27/5 = 5.4 is very close to that found in the SEM micrograph *n*_*c*_/*n*_*b*_ = 32/5 = 6.4, as shown in Figure 5f. This leads to the TDD value of TDD ≈ 3×10^12^ cm^–2^. For the *r*-cut Al_2_O_3_ substrate shown in Figure 8c, the situation is quite similar, but ε_*a*M1_ = ε_*z*_ > 0 and transition M1 → M2 is now possible, and relaxation happens through mechanism D + M2. Here, the strain energy potential anisotropy is at lowest with *n*_*b*_/*n*_*c*_ = 20/7 = 2.9. Furthermore, on the *c*-cut Al_2_O_3_ substrate, shown in Figure 8d, VO_2_ thin film has orientation with ε_*a*M1_ = ε_*x*_ > 0, relaxation occurs with mechanism D + M2, so that here the strain energy potential was calculated against A phase interface layer, i.e., mechanism D + M2 + A, as explained in Figures 5b and 7d in detail. Even though the anisotropy is now at its highest with *n*_*a*_/*n*_*c*_ = 68/5 = 13.6, a clear energy minimum was reached with reasonable residual strain energy values *E*_ε_ ≈ 4 × 10^–3^ GPa. Finally, the reference case of VO_2_ thin film on MgO substrate is shown in Figure 8b, with ε_*a*M1_ = ε_*x*,*y*_ > 0. The relaxation mechanism is now D + M2, and interestingly only M1 phase unit-cell axis *a*_M1_ lays in the thin film–substrate interface plane and thus the strain energy potential minimum is one-dimensional. Note that in this case eq 7 is not directly applicable.

In more general context, presented interface strain-energy potentials can also be utilized to model and design the total effects of the thin-film–substrate interface on the crystal potential, e.g., through the last term of eq 1, on the film side. This concerns especially the anisotropic crystal orientation-dependent defect structures, such as vacancies and dislocations with their electronic properties including charge density, spin coupling, and conductivity. These structures have been found promising candidates, for example, in topological qubit quantum devices.^44^

## 5 Discussion

**Figure 9:**
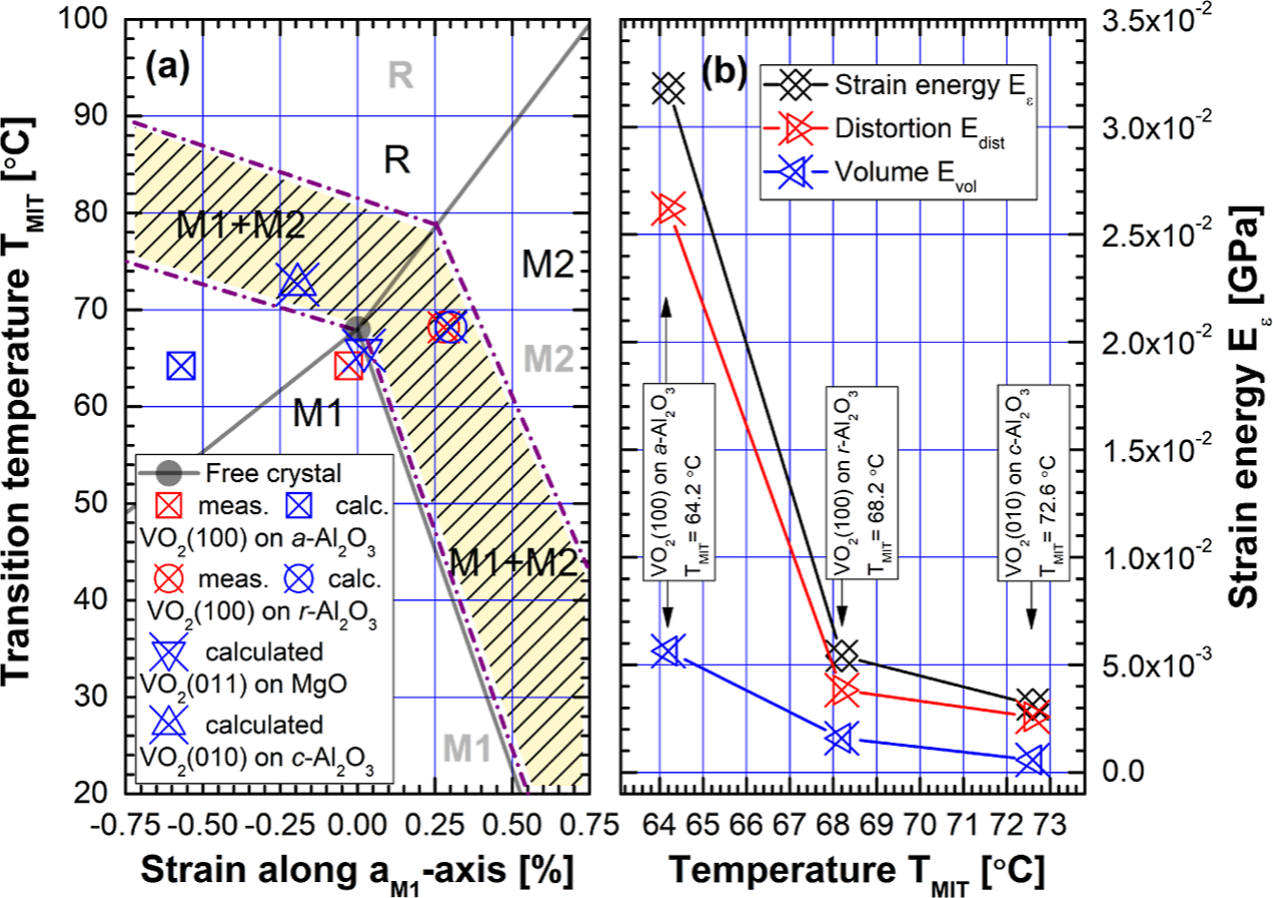
(a) Phase diagram showing a M1 + M2 phase coexistence area appearing in *T*_MIT_(ε_*a*M1_) graph for the mechanically constrained epitaxial VO_2_ thin films. Phase diagram for a single crystalline VO_2_ bulk is depicted for comparison with gray lines and letters M1, M2, and R in the background. (b) The minima of residual misfit strain energy *E*_ε_ values as a function of *T*_MIT_, with the breakdown to the components of volume change energy *E*_vol_, and the symmetry distortion energy *E*_dist_.

Since VO_2_ thin-film structures on the various substrates were found to have deviations in their microstructures, one would assume that their transition temperatures *T*_MIT_ should not follow the linear dependence as a function of M1 phase *a*-axis strain ε_*a*M1_ in comparison to each other, as predicted by the conventional Clausius–Clapeyron model.^39,40^ This is definitely the case in here as well, but still it is informative to study the representative data points *T*_MIT_(ε_*a*M1_) in such a phase diagram graph, as shown in Figure 9a.

Phase diagram for a single crystalline VO_2_ bulk is depicted for comparison with gray lines and letters M1, M2, and R in the background. Phase coexistence of M1 and M2 phases should not appear in the first-order MIT effect of VO_2_ single crystals, but in the mechanically constrained fibers and thin films phase, coexistence is often found.^24,26,45^ In a similar way as Park et al. in,^40^ we propose a modified phase diagram showing a M1+M2 phase coexistence area appearing in the *T*_MIT_(ε_*a*M1_) graph for the mechanically constrained epitaxial VO_2_ thin films, as an obvious conclusion from the presented experimental and model-calculation results, shown as shaded area in Figure 9a. Even if the data points cannot be expected to fall on the same linear conventional Clausius–Clapeyron model due to structural deviations, the values of residual misfit strain energy *E*_ε_ between samples on Al_2_O_3_ are definitely comparable, and are actually revealing interesting properties of the MIT effect. The minima of residual misfit strain energy *E*_ε_ values presented in Figure 7a,c,d as a function of corresponding *T*_MIT_ are presented in Figure 9b, with the breakdown to the components of energy related to volume change *E*_vol_, and the strain energy inducing the distortion of the symmetry *E*_dist_, so that^41^
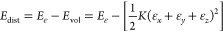
8where *K* = *Y*/[3(1 – 2υ)] stands for the bulk modulus. Equation 8 holds for a linear elastic isotropic material. It can be seen that the residual misfit strain energy *E*_ε_ is inversely proportional to the MIT temperature *T*_MIT_. As the critical disordered interface layer structure with increased complexity emerges to the samples through relaxation mechanisms D, D + M2, and D + M2 + A, the strain energy *E*_ε_ is really minimized. However, as a consequence of the complex defect and phase structure in the relaxed interface layer between the VO_2_ thin film and substrate at RT, the transition back to high-temperature R phase requires now more heat in addition to M1 → R phase transition enthalpy Δ*H* = Δ*H*_M1_ shown in eq 1, so that phase transition enthalpy increases to (Δ*H*_M1_ + Δ*H*_D_), (Δ*H*_M1_ + Δ*H*_D_ + Δ*H*_M2_), and (Δ*H*_M1_ + Δ*H*_D_ + Δ*H*_M2_ + Δ*H*_A_) for the mechanisms D, D + M2, and D + M2 + A, respectively. This explains very well not only the dependence of the residual misfit strain energy *E*_ε_ as a function of *T*_MIT_ but also the observed increase in the hysteresis values with different relaxation mechanisms. Furthermore, it becomes clear from Figure 9b, that over 70% of *E*_ε_ for all samples is in the form of *E*_dist_ inducing the distortion of the symmetry elements, thus promoting the MIT effect to take place in mechanically constrained VO_2_ thin films through the SPT process, as in the Peierls mechanism.

## 6 Conclusions

Misfit strain state phenomena in the interface between technologically important vanadium oxide VO_2_ nanostructured epitaxial thin films and Al_2_O_3_, and MgO single crystalline substrates were studied in detail. Special attention was paid for the misfit strain relaxation mechanisms, and their effects on the MIT effect of VO_2_ thin films. It was found, that misfit strain in the interface is relaxed through three mechanisms including formation of dislocations, polymorphic phase transitions, and their combinations. As a consequence, there is a specific critical interface disorder layer formed in the interface. A phenomenological model for interface Cauchy misfit strain, taking into account the observed relaxation mechanisms, was developed. Obtained model calculations confirmed the experimental results of strain relaxation effects on decreasing transition temperature *T*_MIT_ and increasing hysteresis, together with a strong suggestion, that in the mechanically constrained VO_2_ thin films, the MIT effect is driven by a SPT phenomenon through Peierls mechanism.
